# Actual Cautery in the Treatment of Cartilaginous Quittor

**Published:** 1901-03

**Authors:** S. J. J. Harger

**Affiliations:** Veterinary Department, University of Pennsylvania


### ACTUAL CAUTERY IN THE TREATMENT OF CARTILAGINOUS QUITTOR.

By S. J. J. Harger, V.M.D.,
VETERINARY DEPARTMENT, UNIVERSITY OF PENNSYLVANIA.

As in tetanus, in cartilaginous quittor of the third phalanx all possibly suitable remedies in the pharmacopœia have been appropriated for its treatment. Each remedy enjoyed special distinction, among them being sulphate of copper, Villatte's solution, arsenic, corrosive sublimate, tincture of the perchloride of iron. Monsel's solution, and, more recently in France, the resinate of copper.

Ten years ago the operation of Lafosse was much in vogue. Sometimes the excision of the lateral cartilage was only partial, being confined to the removal of the necrosed parts. This can be done with success. Later, Bayer's operation has been practised. Without going into details as to the merits of these operations, suffice it to say that in a great many respects they were found wanting and are at present comparatively rarely performed. Such complications as septic infection of the tissues of the foot, suppuration, separation of the hoof, arthritis, septicæmia, etc., occurred with undesirable frequency.

Caustic injections were again resorted to. I wish to call attention here to the use of the hot iron in such cases. About two years ago I first used this treatment, but have neglected it since. For some months at the University veterinary hospital I have in this manner treated all such cases as came under my notice. If there is much lameness and swelling the wall is thinned and the region of the cartilage then antisepticized with corrosive sublimate solution. The fistula—one or more, as the case may be—is then thoroughly cauterized with the hot iron, care being taken to cauterize well the bottom of the tract. All the necrosed tissue should thus be destroyed and the dry eschar should be continuous all around with healthy tissue only. If the fistula is not too deep I use the point of the thermocautery. In lieu of this any sharp-pointed piece of iron will answer the purpose. An antiseptic compress is then applied and the dressing may be removed as often as necessary. Under the eschar a granulating surface will form and the fistula will soon close up and cicatrize. Over the posterior two-thirds of the cartilage the deep cauterization of fistulous tracts is not attended with much danger, while in the anterior third care must be employed not to perforate the lateral ligament or the lateral synovial cul-de-sac of the last interphalangeal articulation. With the use of the hot iron the depth of the cauterization can be regulated, while with other caustic agents the slough is often more extensive than desired.

The rationale of this treatment is very evident. In spite of the numerous bloodvessels over the periphery of the lateral cartilage, its intrinsic bloodvessel system is not sufficiently developed to fight successfully against the pathogenic micro-organisms of the atmosphere; hence its necrosis when exposed to the air. It will, however, recover from traumatisms, such as lacerating wounds or partial ablation, if not contaminated with atmospheric germs. This is how the eschar acts. The hot iron destroys the necrosed tissue and forms a bacteria-proof coating under which the healing process continues favorably. I am not prepared to give a tabulated account of cases, but the results have been exceedingly satisfactory. One of the cases recovered in two weeks, but usually a little more time is required. The fistulous tract soon assumes the aspect of a healthy ulcer and the cavity soon fills up and cicatrizes. The service of the horse is not materially interfered with. If a fistula refuses to heal it indicates that not all of the necrosed tissue has been destroyed and the cauterization must be repeated. I now take charge of a case of quittor with a degree of self-assurance not to be compared with that experienced when cases were treated for months and then only with uncertain results.

